# Effect of mindfulness yoga on anxiety and depression in early breast cancer patients received adjuvant chemotherapy: a randomized clinical trial

**DOI:** 10.1007/s00432-022-04167-y

**Published:** 2022-07-05

**Authors:** Weimin Liu, Juan Liu, Lan Ma, Jing Chen

**Affiliations:** 1 School of Nursing, Capital Medical University, No. 10 You-an-men Wai Xi-tou-tiao, Feng-Tai District, Beijing, 100069 China; 2 Department of Orthopaedic Surgery, Beijing Shijitan Hospital Affiliated to Capital Medical University, No. 10 Tieyi Road Yang-fang-dian, Hai-Dian District, Beijing, 100038 China; 3 Department of Breast Surgery, Beijing Shijitan Hospital Affiliated to Capital Medical University, No. 10 Tieyi Road Yang-fang-dian, Hai-dian District, Beijing, 100038 China; 4 Party Committee Office, Beijing Shijitan Hospital Affiliated to Capital Medical University, No. 10 Tieyi Road Yang-fang-dian, Hai-Dian District, Beijing, 100038 China

**Keywords:** Breast cancer, Anxiety, Depression, Mindfulness yoga, Adjuvant chemotherapy

## Abstract

**Purpose:**

The objective of this study is to explore the effects of a mindfulness yoga treatment on emotional disorders, fatigue, pain, and health-related quality of life in early-stage breast cancer patients.

**Methods:**

The eligible 136 participants were randomly assigned 1:1 to the experimental group (mindfulness yoga + conventional care) and the control group (conventional care). The hospital anxiety and depression scale was used to assess anxiety and depression symptoms as the primary outcome. Secondary results comprised fatigue (RPFS-CV), pain (BPI-C), and health-related quality of life (FACT-B). Assessments were performed at baseline time, the 8th week, and the 20th week.

**Results:**

The Experimental group had a better prognosis in comparison with those in the control group, especially for anxiety [inter-group effect, *T*1: 1.18 (95% CI 0.20–2.17; *P* = 0.018)], depression [*T*1: 1.49 (95% CI 0.48–2.50; *P* = 0.004)] and health-related life quality [*T*1: − 6.34 (95% CI − 11.81 to − 0.87; *P* = 0.023)]. While fatigue [*T*1: 0.23 (95% CI − 0.24–0.69; *P* = 0.337); *T*2: 0.27 (95% CI − 0.16–0.71; *P* = 0.219)] and pain [*T*1: 1.11 (95% CI − 0.05–2.27; *P* = 0.060); *T*2: 0.68 (95% CI − 0.27–1.62; *P* = 0.159)] were not different between the two groups.

**Conclusion:**

In patients with early-stage breast cancer who had received adjuvant chemotherapy, treatment with mindfulness yoga is as effective as conventional care in improving physical function. Along with other treatments, mindfulness yoga may help alleviate anxiety and depression to improve the overall physical and mental health and quality of life of early-stage breast cancer patients.

**Chinese Clinical Trial Registry Registration number**: ChiCTR2100052842, Reg. Date: 2021/11/6.

## Introduction

Breast cancer is the most widely known cancer in females around the world with a constantly increasing incidence rate (Sung et al. 2021). Early-stage breast cancer is considered curable, and current clinical treatment includes surgery, systemic chemotherapy, local radiotherapy, and endocrine therapy (Harbeck and Gnant 2017). Cancer and its treatment can result in complications and side effects like gastrointestinal dysfunction, fatigue, pain, emotional disorders, etc. (Hill et al. 2011; Pearce et al. 2017; Khan et al. 2020). Emotional disorders, including anxiety and depression, are very common in breast cancer patients, with about 50% of women suffering from early-stage breast cancer dealing with anxiety, depression, or both within one year of diagnosis (Burgess et al. 2005), which is associated with other chronic illnesses, lack of social support (Jimenez-Fonseca et al. 2018; Yan et al. 2019), financial difficulties, physical and cognitive decline (Gray et al. 2014). Evidence showed that anxiety and depression are significantly correlated with reduced life quality in breast cancer patients (Nipp et al. 2016; Phoosuwan and Lundberg 2022). Although these emotional disorders are prevalent and pose a serious threat to women's physical and mental health, they have rarely received clinical attention and have been managed ineffectively. First-line pharmacological treatment of emotional disorders in breast cancer patients, such as selective serotonin reuptake inhibitors (SSRIs), can increase breast cancer mortality (Busby et al. 2018). Therefore, researchers must pay attention to complementary and alternative medicine therapies to manage stress and symptoms in breast cancer patients.

Mindfulness yoga, a routine practice in mindfulness-based stress reduction (MBSR) (Pascoe et al. 2017), is a standardized approach to mind–body exercise that can reduce emotional disturbances by reducing sensory-perceptual judgments and developing a closer connection between consciousness and body. Mindfulness yoga may be particularly helpful for anxiety and depression because it increases stress tolerance. The side effects of cancer treatment on patients (e.g., effect on hair, sexual function, or organs), survival expectations, and effects on work as well as social roles can result in chronic psychological stress. Mindfulness yoga focuses on the interplay between the brain, mind, body, and behavior to use the mind to influence body function and promote wellness. Although mind–body therapies are commonly used by breast cancer patients, and some small-scale studies (Carson et al. 2017; Porter et al. 2019; Zimmaro et al. 2020) have proved their safety, feasibility, and initial benefits, standardized and rigorous methods are needed to assess their clinical effectiveness and mechanisms.

We conducted a randomized, double-blind clinical trial of mindfulness yoga exercise for early-stage breast cancer patients. We hypothesized that mindfulness yoga would alleviate anxiety and depression, fatigue, pain, and quality of life in early-stage breast cancer patients in comparison with conventional care.

## Methods

### Study design

This study is a prospective, single-center, parallel, double-blind, randomized, controlled clinical trial on breast cancer that compares mindfulness yoga with conventional care. The study was carried out following the International Conference on Harmonization Good Clinical Practice Guidelines and the Declaration of Helsinki. This study was approved by the ethical commitment review of Beijing Jitan Hospital, Capital Medical University [sjtky11-1x-2021(96)], and followed the consolidated standards for reporting of clinical trials (CONSORT) reporting guidelines. Written informed consent was given by all participants, and all data were anonymized to keep the identity of participants private.

### Study participants

Patients with primary breast cancer were recruited consecutively from July 28, 2021, to April 21, 2022, from a large tertiary hospital breast surgery department in Beijing. The enrolled participants were then screened according to inclusion and exclusion criteria. Inclusion criteria were: histological diagnosis of primary breast cancer; patients who had undergone mastectomy; patients who had their first postoperative chemotherapy; pathological stage of the tumor was stage I or II; expected survival ≥ 1 year; body mass index (BMI) ≥ 18 kg/m^2^; and signed informed consent to voluntarily enroll in this study. Exclusion criteria were previous history of psychiatric disorders, comorbid other serious life-threatening diseases, showing serious physical disorders such as deafness, inability to answer questions; lymphedema of the upper extremities; receiving other exercise or psychological interventions that might prevent full participation in the study.

### Blinding and randomization

To keep the participants from knowing the experimental and control groups, the recruitment advertisement described the study as "learning mindfulness yoga may be helpful for emotional relief", but showed no content details. This avoided uneven group allocation, differences in group expectations, and selection bias. The data evaluators were unaware of the group assignment.

Eligible participants went through a baseline assessment. Before the trial, random sequences were produced using Stata 16.0 software, and group allocation information was hidden on cards, maintained in airtight, opaque envelopes, and provided to the study coordinator (who did not perform data analysis). The ratio of the experimental group to the control group was 1:1, the envelopes were opened sequentially in order of enrollment sequence of patients, and the patients were given either conventional care or mindfulness yoga according to the random number grouped in the envelope.

### Interventions

#### Mindfulness yoga training

The experimental group received 90 min of weekly orthodox yoga practice on top of regular care over 8 weeks. The mindfulness yoga training method consisted of four basic phases: the first phase of mindfulness meditation (20 min), the second phase of mindfulness yoga postures accompanied by mindfulness breathing guidance (50 min), the third phase of mindfulness yoga body scan (10 min), and the fourth phase of sensory summary and question and answer (10 min). The program was developed based on a review of relevant studies (Kwok et al. 2019) and clinical practice, concerning the mindfulness yoga practice in the mindfulness-based stress reduction (MBSR) and the chapter on mindfulness yoga in the book mindfulness cancer recovery by Linda Carlson and Michael Speca.

### Routine care

Over 8 weeks, the control group received 60 min per week of routine nursing intervention. The program included psychological care; dietary guidance; special nursing; health education; telephone follow-up; and lifestyle guidance, which were examined by clinical nurse specialists to confirm its effectiveness for breast cancer patients.

These interventions were consistent in format (group), frequency (weekly), duration (8 weeks), and site (functional rehabilitation area of the breast department of Beijing Shijitan Hospital). In this study, a combination of three forms of periodic face-to-face interventions, bedside instruction, and personalized coaching on the WeChat platform was used. In this program, all instructors were trained and underwent mindfulness-based stress reduction (MBSR) faculty with more than 10 years of clinical care experience in oncology, including mindfulness yoga training under the guidance of a professional counselor and senior positive yogi. All participants received a manual, and audio and video were only given to participants in the mindfulness yoga group. Participants that considered to have completed the therapy were the ones who attended at least 6 out of 8 practice sessions. Adherence was considered good if participants attended more than 80% of the total number of interventions. In addition, WeChat groups (regular care group and mindfulness yoga group) were established, and regular announcements, sharing of resources, and assignments were made through the WeChat group.

#### Follow-up

The data assessors were trained. Each participant received a face-to-face assessment and interview to minimize bias. All outcome procedures were performed at each time point: baseline (*T*0), 8 weeks post-intervention (*T*1), and 20 weeks post-intervention (*T*2).

#### Wait-list controls

Participants in the control group received routine care, including health education and follow-up care instructions. After the wait-list study, these participants were introduced to the mindfulness yoga program and provided with audio and materials for the intervention.

### Outcome measures

The research team designed their own socio-demographic information. Outcome data were collected using the questionnaire star program.

### Primary outcome

The primary outcome was heart distress in terms of the symptoms of anxiety and depression, using the validated hospital anxiety and depression scale (Johnston et al. 2000) (HADS), a self-report questionnaire consisting of anxiety and depression subscales. The scale contains 14 entries, each of which is rated on a Likert 4-point scale (0–3), with a total score of 0–7 for each dimension being asymptomatic, 8–10 for mild anxiety-depression symptoms, and 11–21 for significant anxiety or depression symptoms. Chinese scholars have Chineseized the scale, and the HADS (Chinese version) scale has a Cronbach's alpha coefficient of 0.88 (Sun et al. 2017), retest reliability of 0.95, and a calibration correlation validity of 0.60, which has good reliability and validity. In addition, anxiety and depression were reported to be the most important psychological factors in early-stage breast cancer patients, and the levels of these two emotions were considered clinically relevant in this study.

### Secondary outcomes

Secondary outcomes included (1) severity of fatigue symptoms, as measured by the Chinese version of the validated Piper fatigue assessment scale (Piper et al. 1998; So et al. 2003) (RPFS-CV), which contains four dimensions of fatigue assessment: behavioral, emotional, perceptual, and cognitive. This scale is a commonly used multidimensional fatigue self-assessment tool in cancer research (Liu et al. 2020). (2) Measured by the validated Chinese version of the brief pain inventory (BPI-C) (Wang et al. 1996), which is the most commonly used assessment scale in measuring the severity of pain symptoms in cancer patients. (3) Health-related quality of life was calculated using the validated Chinese version of the quality of life measurement scale for breast cancer patients (FACT-B) (Wan et al. 2007; Ng et al. 2012), which reaches a composite index score reflecting the disease-specific health-related quality of life scores in the physical, social/family, emotional, functional, and additional concerns dimensions.

### Adverse events

Events of significant discomfort such as pain, injury, etc. were considered adverse that were caused during the intervention, and participants were informed that if they experienced any adverse events related to this study, they should promptly inform the study team and receive systematic inquiries and follow-up.

### Sample size

According to a study on the relationship between mindfulness yoga and anxiety and depression (Kwok et al. 2019), the standard deviation of the two groups with anxiety as the main outcome indicator was reported as *σ*1 = 3.49, *σ*2 = 3.06, *δ* = 1.91, and the standard deviation of the two groups with depression as the main outcome indicator was *σ*1 = 3.65, *σ*2 = 3.18, *δ* = 1.8. assuming power = 80%, alpha = 0.050, using a two-sided test, taking into account a 20% attrition rate (using PASS statistical software), our recruitment target was a 2-arm trial with 68 participants per arm (*n* = 136).

### Statistical analysis

The SPSS 24.0 statistical software (IBM Corporation) was used to carry out the statistical analysis. Intention-to-treat analyses was accomplished by adding all the available studies to it. Quantitative data, satisfying a normal distribution, were described by $\overline{X }$±*S,* and comparisons between two groups were made by the two independent samples *t*-test, the test statistic was set as *t*. Qualitative data were described by *n* (%), and comparisons between groups were done by the chi-square test or Fisher's exact probability method test, the test statistic was set as *χ*2. Ordinal data were described by *n* (%), and comparisons between two groups were done by the grouped rank-sum test, the test statistic being *z*. All participants were assessed for psychological distress, fatigue symptoms, pain, and changes in quality of life at *T*0, *T*1, and *T*2. We used generalized estimating equation models with change at baseline examination as the dependent variable and group, time, and the interaction of group time as the independent variables. The approximate normality of each outcome and change in them was confirmed by the test. Baseline values were added as covariates to ensure that statistical balance was not captured at random and, more importantly, to lower the error variance. We used a stratified gate-keeping strategy, first testing the interaction terms, then testing for group effects, time effects if significantly different, and then testing to calculate main effects if not significant. All tests were carried out with a two-tailed test with a test level of *α* = 0.05, and all differences were statistically significant at *P* ≤ 0.05.

## Results

**Fig. 1:**
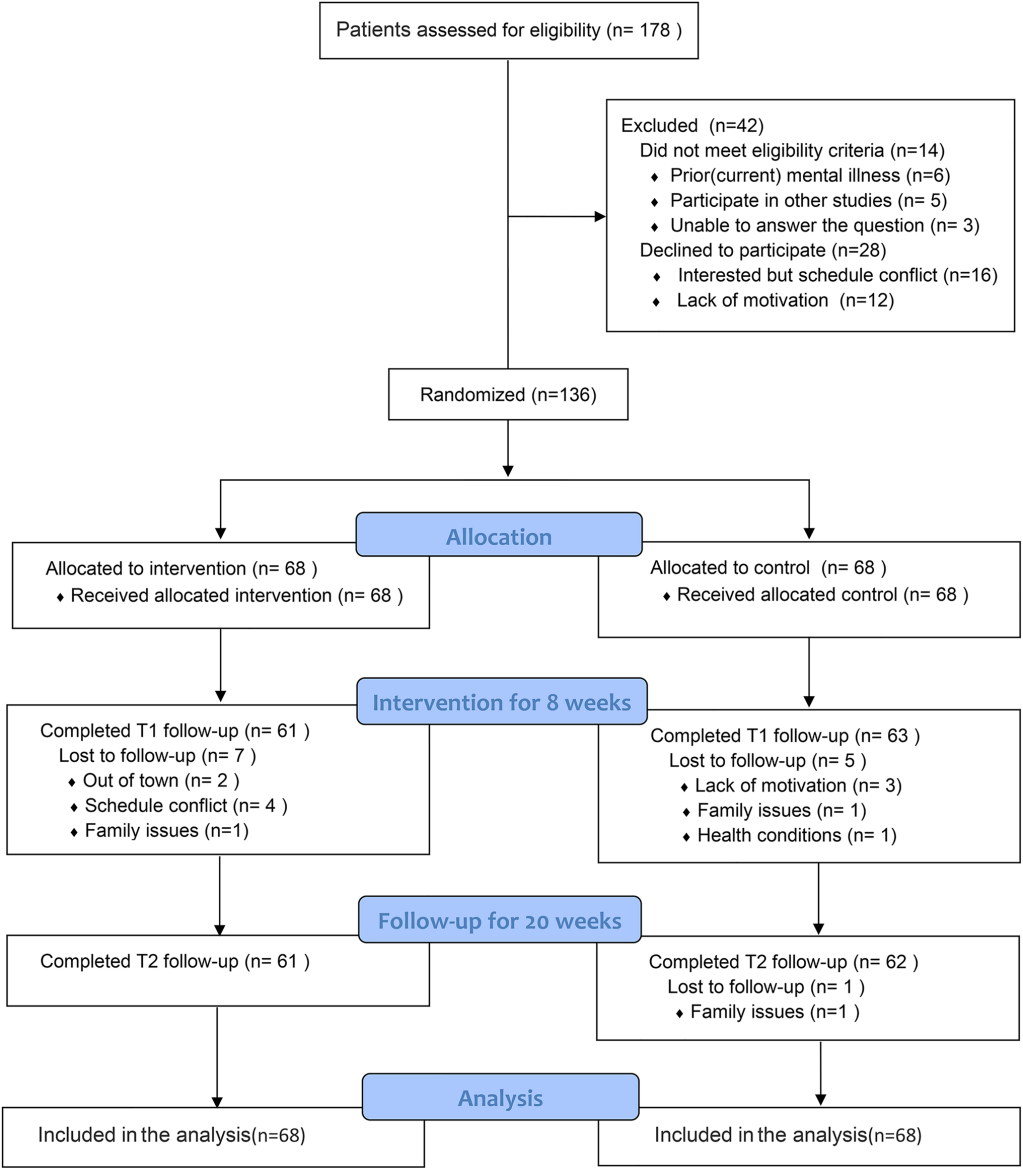
The research framework of this study

Of the total 178 screened potential participants, 14 were not eligible as per the eligibility criteria and 28 declined to participate (registration rate: 76.4%) (Fig. 1). Among the 136 participants randomly assigned, 68 were in the control group and 68 were in the experimental group. Sixty-one (89.7%) of the 68 participants randomly assigned to the experimental group participated in at least 6 exercises, while 63 (92.6%) of the 68 participants randomly assigned to the control group participated in at least 6 exercises.

During the intervention period, good compliance was 82.3% (56/68) of the experimental group and 80.9% (55/68) of the control group. 12 (8.8%) of the 136 participants in the T1 period dropped out, including 7 of 68 (10.3%) in the experimental group; 5 of 68 (7.4%) in the control group. 1 of 136 (control group: 0.7%) dropped out.

### Participant characteristics

The experimental and control groups were similar in terms of sociodemographic and clinical traits (Table 1). Participants were all female, 51–60 (*n* = 48, 35.29%) years old were the most numerous, mean BMI was 24.20, and most (*n* = 100, 73.53%) participants lived in urban areas. The differences in age, BMI, ethnicity, marital status, occupation, education, payment method, residence, surgical procedure, pathological stage, and chemotherapy regimen between the two groups of participants were not statistically significant (*P* ≥ 0.05) and were comparable.

**Table 1:** Baseline sociodemographic characteristics of participants

Baseline characteristic	No. (%)			*t*/*z*/$\chi^{2}$	*P*
	Control (*n* = 68)	Experimental (*n* = 68)	All (*n* = 136)		
BMI, $\overline{x} \pm s$	23.69 ± 3.16	24.71 ± 3.69	24.20 ± 3.46	− 1.727	0.086
*Age*				− 1.118	0.264
18–30	2 (2.94)	1 (1.47)	3 (2.21)		
31–40	9 (13.24)	11 (16.18)	20 (14.71)		
41–50	16 (23.53)	19 (27.94)	35 (25.74)		
51–60	22 (32.35)	26 (38.24)	48 (35.29)		
> 60	19 (27.94)	11 (16.18)	30 (22.06)		
*Nationality*				2.119	0.808
Han	65 (95.59)	64 (94.12)	129 (94.85)		
Hui	1 (1.47)	1 (1.47)	2 (1.47)		
Man	1 (1.47)	3 (4.41)	4 (2.94)		
Minority	1 (1.47)	0 (0.00)	1 (0.74)		
*Marital status*				1.501	0.855
Unmarried	2 (2.94)	2 (2.94)	4 (2.94)		
Married	63 (92.65)	65 (95.59)	128 (94.12)		
Divorced	2 (2.94)	1 (1.47)	3 (2.21)		
Widowed	1 (1.47)	0 (0.00)	1 (0.74)		
*Occupation*				0.040	1.000
In-service	21 (30.88)	20 (29.41)	41 (30.15)		
Unemployed	15 (22.06)	15 (22.06)	30 (22.06)		
Retired	32 (47.06)	33 (48.53)	65 (47.79)		
*Education degree*				− 0.713	0.476
Junior high school	23 (33.82)	23 (33.82)	46 (33.82)		
Senior high school	24 (35.29)	17 (25.00)	41 (30.15)		
Junior college	11 (16.18)	15 (22.06)	26 (19.12)		
Bachelor	9 (13.24)	11 (16.18)	20 (14.71)		
Master's and above	1 (1.47)	2 (2.94)	3 (2.21)		
*Payment method*				0.522	1.000
Self pay	7 (10.29)	6 (8.82)	13 (9.56)		
Medical insurance	60 (88.24)	60 (88.24)	120 (88.24)		
Public-fee	1 (1.47)	2 (2.94)	3 (2.21)		
*Place of residence*				0.151	0.846
Urban	51 (75.00)	49 (72.06)	100 (73.53)		
Rural	17 (25.00)	19 (27.94)	36 (26.47)		
*Operation way*				3.498	0.194
Breast conserving surgery	16 (23.53)	9 (13.24)	25 (18.38)		
Improved radical surgery	17 (25.00)	25 (36.76)	42 (30.88)		
Radical mastectomy	35 (51.47)	34 (50.00)	69 (50.74)		
*Pathological stage*				− 1.794	0.073
I	21 (30.90)	12 (17.60)	33 (24.30)		
II	47 (69.10)	56 (82.40)	103 (75.70)		
*Chemotherapy regimens*				7.147	0.308
AC	14 (20.59)	9 (13.24)	23 (16.91)		
TC	17 (25.00)	26 (38.24)	43 (31.62)		
EC	1 (1.47)	13 (19.12)	24 (17.65)		
TCH	1 (1.47)	4 (5.88)	5 (3.68)		
TA	12 (17.65)	9 (13.24)	21 (15.44)		
TH	7 (10.29)	3 (4.41)	10 (7.35)		
TAC	6 (8.82)	4 (5.88)	10 (7.35)		

*BMI* body mass index

### Primary outcomes

**Fig. 2:**
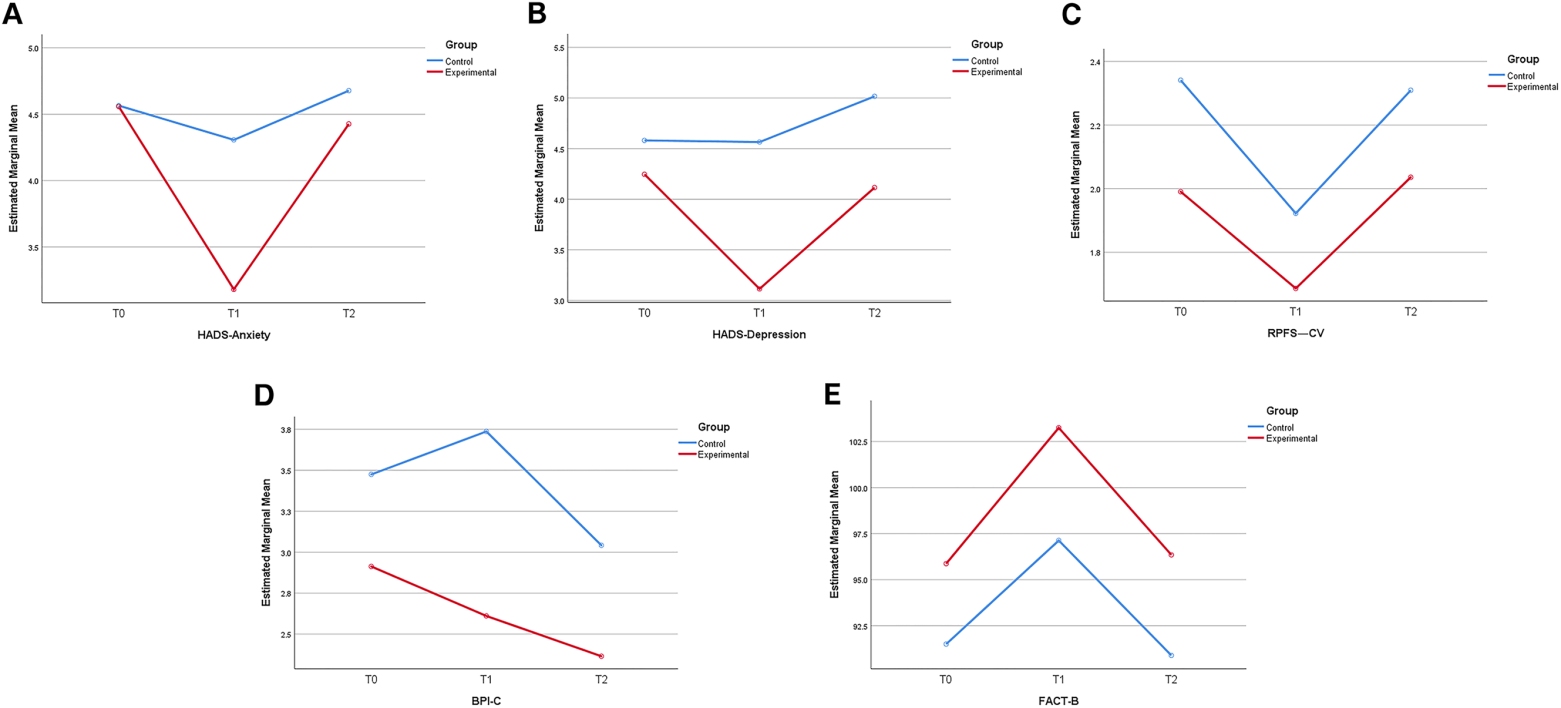
Change of each time factor. **A**–**E** Change trends of anxiety and depression (HADS), fatigue (RPFS-CV), pain (BPI-C), and health-related quality of life (FACT-B) scores at *T*0, *T*1, and *T*2 in the two groups

For anxiety and depressive symptoms, there was a considerable variation between groups at *T*1 (*P* = 0.018; *P* = 0.004) for participants in both groups. Compared to the control group, the experimental group showed more significant remission of anxiety [*T*1: 1.18 (95% CI 0.20–2.17; *P* = 0.018)] and depressive symptoms [*T*1: 1.49 (95% CI 0.48–2.50; *P* = 0.004)]. No statistical difference was observed between the groups at T2 for participants in both groups [anxiety: 0.25 (95% CI − 0.67 to 1.17; *P* = 0.594); depression: 0.90 (95% CI − 0.09 to 1.90; *P* = 0.076)]. In the control group, we did not observe a noticeable improvement in the symptoms of anxiety and depression at any time point (Fig. 2).

### Secondary outcomes

For the health-related quality of life (FACT-B), a major variation between the groups at *T*1 (*P* = 0.023) was observed for participants in both groups. The health-related quality of life score was significantly higher in the experimental group compared to the control group [*T*1: − 6.34 (95% CI − 11.81 to − 0.87; *P* = 0.023)]. There was no statistically significant difference between the groups at *T*2 two groups of participants [− 5.46 (95% CI − 11.03 to 0.12; *P* = 0.055)]. For time effects, there was a significant within-group difference between the experimental and control groups at *T*2 compared to *T*1 (control: *P* < 0.001; test: *P* < 0.001). According to fatigue [*T*1: 0.23 (95% CI − 0.24 to 0.69; *P* = 0.337); *T*2: 0.27 (95% CI − 0.16 to 0.71; *P* = 0.219)] and pain [*T*1: 1.11 (95% CI − 0.05 to 2. 27; *P* = 0.060); *T*2: 0.68 (95% CI − 0.27 to 1.62; *P* = 0.159)] scores did not show significant between-group differences, however, fatigue (RPFS-CV) scores at *T*1 decreased from baseline for participants in both groups. Pain (BPI-C) scores at *T*2 decreased from baseline for participants in both groups, (Fig. 2; Table. 2).

**Table 2:** Generalized estimating equation analysis for the comparison of outcomes

Instrument		*T*0 (*n* = 136)	*T*1 (*n* = 124)	*T*2 (*n* = 123)	Wald Chi-square	*P*
HADS-anxiety	Control	4.62 ± 2.96	4.37 ± 2.89	4.68 ± 2.46	1.548	0.213
	Experimental	4.56 ± 2.98	3.18 ± 2.76	4.43 ± 2.80	28.133	< 0.001*
	LS mean (95% CI)	0.06 (− 0.97, 1.10)	1.18 (0.20, 2.17)	0.25 (− 0.67, 1.17)		
	Wald Chi-square	301.447	216.258	373.153		
	*P*	0.907	0.018*	0.594		
HADS-depression	Control	4.57 ± 2.80	4.6 ± 3.20	5.02 ± 2.68	1.232	0.267
	Experimental	4.25 ± 3.28	3.11 ± 2.57	4.11 ± 2.98	21.888	< 0.001*
	LS mean (95% CI)	0.33 (− 0.74, 1.39)	1.49 (0.48, 2.50)	0.90 (− 0.09, 1.90)		
	Wald Chi-square	263.847	209.848	317.061		
	*P*	0.549	0.004*	0.076		
RPFS-CV	Control	2.31 ± 0.15	1.91 ± 0.17	2.31 ± 0.14	4.765	0.092
	Experimental	2.07 ± 0.17	1.69 ± 0.17	2.04 ± 0.17	10.845	0.004*
	LS mean (95% CI)	0.24 (− 0.21, 0.69)	0.23 (− 0.24, 0.69)	0.27 (− 0.16, 0.71)		
	Wald Chi-square	1.069	0.921	1.513		
	*P*	0.301	0.337	0.219		
BPI-C	Control	3.47 ± 0.40	3.72 ± 0.43	3.04 ± 0.38	6.092	0.048*
	Experimental	3.13 ± 0.37	2.61 ± 0.40	2.36 ± 0.30	8.914	0.012*
	LS mean (95% CI)	0.30 (− 0.76, 1.36)	1.11 (− 0.05, 2.27)	0.68 (− 0.27, 1.62)		
	Wald Chi-square	0.309	3.534	1.981		
	*P*	0.579	0.060	0.159		
FACT-B	Control	91.4 ± 16.68	96.9 ± 15.96	90.89 ± 15.74	12.925	< 0.001*
	Experimental	95.87 ± 16.06	103.25 ± 15.37	96.34 ± 16.05	20.937	< 0.001*
	LS mean (95% CI)	− 4.47 (− 10.19, 1.24)	− 6.34 (− 11.81, − 0.87)	− 5.46 (− 11.03, 0.12)		
	Wald Chi-square	4038.735	4929.180	4209.628		
	*P*	0.125	0.023*	0.055		

* *P* ≤ 0.05 indicated statistically significant difference

### Adverse events

One participant (1.4%) reported temporary mild knee pain that related to the practice and was relieved by bedding a thick blanket during practice, and no serious adverse events were reported. One participant (1.4%) reported temporary lower extremity weakness that related to the practice, which was relieved by a 20-min rest during practice, and no serious adverse events were reported.

## Discussion

The current research is consistent with previous studies (Hoffman et al. 2012; Shao et al. 2021) that demonstrated the superiority of the experimental group over the control group in the management of anxiety and depressive symptoms at *T*1, and the mindfulness yoga group was considered to be statistically and clinically significant for improving anxiety and depressive symptoms. Most breast cancer survivors adapt well to life after cancer, but some experience persistent anxiety or depression, and for women newly diagnosed with breast cancer, mental health problems are particularly prevalent. These mood disorders may not meet clinical diagnostic criteria, but subclinical symptoms can affect quality of life. With limited resources and access to traditional medical and psychological care, effective evidence-based strategies need to be developed to reduce the duration of attacks, prevent recurrence and potentially reduce the need for conventional care. Our research demonstrates the effectiveness of mindfulness yoga, in particular, its popularity and appeal to many people because of its broad focus on mind–body or lifestyle interventions as a way to promote physical and mental health, rather than just treat mental illness. As a lifestyle adjunct, mindfulness yoga can be targeted at cancer patients who have specific concerns, such as their reluctance to use medication. However, we found that the effect of mindfulness yoga on psychological outcomes changed over time, with the group differences at *T*1 disappearing at *T*2 when both groups showed an unambiguous decline. This is consistent with the results of positive cognitive therapy to improve mental, physical, and spiritual domains of well-being in breast cancer patients in the same population (Haller et al. 2017; Park et al. 2020; Chang et al. 2021), in which treatment effects declined at follow-up. This suggests that interventions based on mindfulness demand continuous practice and provide patients with skills for effective long-term emotional and symptom management. There is evidence (Modica and Hoenig 2018) that mindfulness-based practice is an effective intervention for reducing fear of recurrence, anxiety, and depressive symptoms in breast cancer patients. These patients exhibit higher levels of physical and psychological symptoms due to fear of relapse and they rarely participate in screening and follow-up. However, since the improvement in symptoms related to anxiety and depression often disappears in the short term after the intervention, regular participation in interventions with mindfulness exercises is also recommended to keep the learned skills up to date. On the other hand, for some patients with early-stage breast cancer, the intensive clinical treatment phase following the initial diagnosis delays their psychological adjustment to the cancer diagnosis until the end of treatment, when patients may begin to consider the importance of potentially life-threatening events and adjust to long-term changes in the body, at which point anxiety and depression may emerge. All participants enrolled in this study were discharged after completing six cycles of adjuvant chemotherapy during *T*2 follow-up, and for these participants, discharge from a cancer treatment facility may represent an abrupt cessation of routine support, and this perceived abandonment may raise anxiety and depression levels to diagnostic thresholds. Breast cancer is the most common non-skin cancer affecting women around the globe, with more than 90% of tumors diagnosed as carcinoma in situ or confined to the breast or regional lymph nodes (Greenberg et al. 2011). Therefore, it is necessary to provide physical and mental exercise programs to improve psychological symptoms in this particular group of early breast cancer patients. This is consistent with previous studies (Shneerson et al. 2013) where patients with early breast cancer benefited from complementary and alternative medicine during chemotherapy. Consistent with recent studies (Carlson et al. 2013; Lengacher et al. 2018), we accurately hypothesized a statistically significant and clinically meaningful relationship between mindfulness yoga and health-related quality of life, with participants in the experimental group showing greater improvements in health-related quality of life (FACT-B) scores at *T*1 compared to the control group. We hypothesized a greater effect of mindfulness yoga on fatigue and pain in patients with early-stage breast cancer, but participants in both groups showed improvements in fatigue and pain without statistically and clinically significant advantages. This may help drive the mechanism of clinical improvement, and the provision of a supervised mindfulness-based exercise program (mindfulness walking and mindfulness yoga) on top of conventional care during adjuvant treatment of early breast cancer, and may be considered for future implementation in the rehabilitation of early-stage breast cancer patients. In conclusion, mindfulness-based exercise provides a plethora of health benefits for patients with early-stage breast cancer, ranging from enhanced physical function and quality of life to improved psychosomatic symptoms including anxiety and depression. Given that psychological symptoms such as anxiety and depression are indirectly associated with increased overall morbidity and mortality (Meier et al. 2016), the practice of mindfulness therapy does appear to be particularly important as adherence to follow-up care and lifestyle issues deserve close attention.

Our study highlighted that only the experimental group indicated great improvements in the psychological aspects of early breast cancer patients. It was shown that mindfulness yoga is more effective in comparison with conventional care in managing emotional disturbances. The remarkable results of the mindfulness yoga program in improving mental health successfully confirm that mindfulness in the intervention is a positive element.

Mindfulness is an awareness type that emerges by purposefully focusing attention on the present moment and becoming aware, without judgment, of the experiences that present themselves moment by moment. All kinds of mindfulness therapies are centered on the concept of mindfulness and rely on various forms of movement such as walking, meditation, and various forms of movement to perceive human mental activities without any judgment, analysis, reaction, or evaluation, but simply to feel, notice, and recognize. Therefore, early breast cancer patients who engage in mindfulness exercises inevitably show more spiritual-psychological self-care, enhancing their greater acceptance of difficulties and facing the various side effects and complications that come during cancer treatment with greater calmness.

These findings are consistent with recent studies that have shown the beneficial effects of mindfulness-based interventions on the physical and mental health of patients with chronic disease (Cavalera et al. 2016; Farver-Vestergaard et al. 2018), Through mindfulness-based exercises, patients understand how to relate their physical symptoms in a non-judgmental manner and in a different way, so that when new symptoms arise, the results seem less disturbing. In addition to its effect on the functional capacity of the body, mindfulness yoga seems to be a beneficial approach to emotional management in early breast cancer patients. Increasing research suggests that this item has implications not only for psychological variables but also for biomarkers (Sanada et al. 2017). To complement the subjective self-reported results of this study on emotional disorders, future studies should incorporate the use of objective psychoneuroimmune markers (e.g., IL-6, TNF-α, ACTH) to elucidate the mediating role of mindfulness yoga concerning stress and inflammatory responses associated with early breast cancer disease progression. Studies are required to assess the long-term advantages and adherence of mindfulness yoga to find reasons for nonadherence and determine minimum requirements. In addition, a cost-benefit analysis is essential. In contrast to other more established mindfulness practices (MBSR and MBCT), a dynamic approach is followed by our mindfulness yoga program for a mindfulness practice that relies on physical activity to gain physiological and psychological aids. Further studies are required for comparing a variety of approaches to mindfulness practice, e.g., movement-oriented yoga versus meditation-oriented mindfulness, to allow patients to choose the best practice to optimize benefits, satisfaction, adherence, and sustainability for each patient.

### Strengths and limitations

The advantages of this research comprise blinding of participants and evaluators of the data, a robust randomized controlled design, and low dropout rate, multiple time points to detect clinical effects of the intervention, and a comprehensive measure of psychophysiological outcomes. Our mindfulness yoga program is tailored to the needs of breast cancer patients and appears to have been taken in by Chinese breast cancer patients.

It is important to acknowledge the limitations of this study. The presence of between-group contamination may be a result of the fact that the participants in this study were all sourced from the same ward of breast surgery at a large tertiary hospital in Beijing, and the two groups differed in their interventions. Although the time and number of classes per week were consistent between the two groups, additional limitations included a commitment to participate in the intervention and scheduling challenges and were a deterrent for many participants who did not have time or were interested in a face-to-face intervention over an 8-week period, which may have limited availability as the study was limited to those who had time and availability. In addition, we intentionally excluded breast cancer patients with severe exercise limitations, and given that the participants in this study were Asian and educated, the results of the study were based on the specific population that participated in the follow-up. All of these characteristics may limit the study's generalizability to the full breast cancer population. It will be important for future studies to evaluate its effects in a more diverse population.

## Conclusions

When the use of first-line medications for emotional disorders can increase breast cancer mortality, it is critical to find non-pharmacological treatments to prevent this phenomenon. Once we learn it, mindfulness yoga can be practiced anywhere at any time, and it is a potentially practical life skill that can be used to deal with a wide range of health issues and distress. Considering current events, anxiety, and depressive episodes may be exacerbated in early-stage breast cancer patients due to the immense stress and anxiety caused by the COVID-19 pandemic, mindfulness yoga may seem particularly useful. In conclusion, mindfulness yoga may help to alleviate anxiety and depression in order to improve the overall physical and mental health of early-stage breast cancer patients.

## Data Availability

The datasets generated during and/or analyzed during the current study are available from the corresponding author on reasonable request.
